# The predictive value of newborn and infant lung ultrasound score for mechanical ventilation needs: a systematic review and meta-analysis

**DOI:** 10.3389/fped.2025.1642202

**Published:** 2025-10-02

**Authors:** Taomei He, Li Ma, Fei Chen

**Affiliations:** Department of Ultrasound Medicine, The First Hospital of Lanzhou University, Lanzhou, Gansu, China

**Keywords:** pulmonary, ultrasound score, infant, mechanical ventilation, meta-analysis

## Abstract

**Objective:**

To investigate the predictive value of neonatal and infant lung ultrasound scores for the need for mechanical ventilation through meta-analysis.

**Methods:**

Literature up to October 1, 2024, on neonatal and infant lung ultrasound scores and mechanical ventilation was searched in PubMed, Web of Science, Embase, and The Cochrane Library databases. The diagnostic accuracy of the included studies was evaluated using the Quality Assessment tool for Diagnostic Accuracy Studies. Revman5.4 and StataSE-64 software were employed to calculate the pooled sensitivity, specificity and AUC value of neonatal and infant lung ultrasound scores for predicting the need of mechanical ventilation.

**Results:**

The meta-analysis comprised 9 studies (7 prospective studies and 2 retrospective), including a total of 1,746 patients. The LUS score predicted the need for mechanical ventilation. Overall sensitivity, specificity, positive likelihood ratio, negative likelihood ratio and diagnostic odds ratio were 74% (95% CI: 66%–81%), 81% (95% CI: 71%–87%), 3.8 (2.4–6.1), 0.32 (0.23–0.46) and 12 (5–6), respectively. The forest plots indicated significant heterogeneity for sensitivity (*p* = 0.81, *I*^2^ = 82.36%, 95%CI: 72.82%–91.90%) and specificity (*p* = 0.74, *I*^2^ = 51.27%, 95%CI: 17.74%–84.80%).

**Conclusion:**

Meta-analysis of multivariate categorical variables indicated that the higher the LUS scores, the greater the risk of mechanical ventilation. The combined results of meta-analysis of diagnostic data suggest that LUS score has high accuracy in predicting the need for mechanical ventilation.

**Systematic Review Registration:**

https://www.crd.york.ac.uk/PROSPERO/, PROSPERO CRD420251029542.

## 1 Introduction

Respiratory diseases are a leading contributor to respiratory failure and death in neonatal inpatient wards (1). There are many causes of respiratory distress in neonates, the most being respiratory distress syndrome (RDS), pneumonia, transient tachypnea of the newborn (wet lung), persistent pulmonary hypertension of the newborn (PPHN), asphyxia, and early-onset sepsis, among others (2). When severe respiratory distress occurs clinically, mechanical assisted ventilation may be required. In developed countries, the use of mechanical ventilation for very preterm infants (<28 weeks) is 80%–90% (3). About 30%–50% of neonates in NICUs in developed countries require mechanical ventilation (4). In developing countries, the proportion of neonates requiting mechanical ventilation is about 10%–20%, but the mortality rate of respiratory failure is as high as 30%–50% (5). The mechanical ventilation rate of very low birth weight infants (<1,500 g) is about 60%–80%. The proportion of mechanical ventilation in full-term infants is about 2%–5% (6, 7). Early identification of severe respiratory failure, improving awareness of the need for mechanical ventilation, and reducing its overall use have become critical areas of clinical focus.

The influencing factors for mechanical ventilation include prematurity, perinatal asphyxia, infection, and maternal pregnancy diseases (8). Currently, identifying key indicators for mechanical ventilation, such as increased respiratory distress (RD), increased fractional inhaled oxygen (FiO_2_), and abnormal gas exchange, has limitations. To illustrate, increases in FiO2 and respiratory severity scores [e.g., Silverman Anderson score (SAS)] often occur only after changes in pulmonary ventilation (9). In addition, arterial blood gas analysis is performed only infrequently because of its invasive nature, whereas capillary samples, frequently used, are cornerstones of decision-making in many NICUs. No reliable indicators currently exist to determine or predict the need for mechanical ventilation (10), underscoring the need for an early-stage screening tool to identify infants requiring invasive ventilation.

The neonatal Lung Ultrasound Score (LUS) is a noninvasive bedside-ultrasound tool that evaluates neonatal pulmonary lesions by quantifying several characteristic signs. The absence of A-lines indicates a loss of normal aeration. Coalescent B-lines suggest pulmonary edema, whether cardiogenic or associated with acute respiratory distress syndrome (ARDS) (11, 12). Lung consolidation indicates alveolar filling (pneumonia, atelectasis). Regional score differences reflect ventilation/perfusion imbalance (13). Pleural effusion can exacerbate atelectasis, further compromising ventilation. The LUS score is typically calculated by dividing the lungs into multiple anatomical regions, each scored from 0 to 3 based on ultrasound findings. A higher total score indicates more severe lung involvement. Its advantages include real-time imaging, absence of radiation and reproducibility, making it especially suitable for dynamic evaluation of critically ill neonates in the neonatal intensive care unit (NICU). LUS quantifies lung ventilation status and severity of lesions by analyzing ultrasound image characteristics of different areas of the lung to provide basis for clinical intervention (14). Evidence from previous studies indicates that pulmonary ultrasound (LU) enables detection of pulmonary ventilation changes before subsequent elevation of RD and FiO_2_ (15). Pulmonary ultrasound offers significant advantages in neonatal intensive care due to its non-radiation, high observer correlation and short learning curve. The classical method of lung region partitioning divides the lungs into 12 regions (6 per side). The anterior chest is divided into upper and lower regions by the intermammillary line. The lateral thorax (from the anterior to posterior axillary lines) is also divided into upper and lower regions. The posterior thorax is similarly separated into upper and lower regions. In some studies, a simplified method is used, dividing each lung into 3 zones (anterior, lateral, posterior), for a total of 6 zones, which is suitable for rapid assessment. Each region is scored based on its ultrasound findings (0–3 points), and the regional scores are summed. The total LUS ranges from 0 to 36 points (12-zone method) or 0–18 points (6-zone method). Vc, et al. showed that a low LUS value can exclude the need for mechanical ventilation (16).

The previous Meta was published by Abdul Razak et al. in 2019 and included literature up to October 2018. Since then, nearly 100 new studies have been published. At present, there are still many controversies in the research, and so far, there is no definitive conclusion on whether LUS can be used to accurately predict mechanical ventilation in newborns and infants. Therefore, our study incorporates the most recently published findings and aims to summarize the existing clinical evidence through a systematic review and meta-analysis.

## 2 Materials and methods

This systematic review and meta-analysis was conducted in accordance with the PRISMA 2020 statement and registered in the PROSPERO database (CRD420251029542).

### 2.1 Literature search strategy

Relevant studies were identified through a systematic literature search on lung ultrasound scores in newborns and infants, from the inception of the databases until October 2024, using PubMed, the Cochrane Library, and Embase. The following MeSH search terms were used: ((("Infant, Newborn"[Mesh]) OR (((((Infants, Newborn) OR (Newborn Infant)) OR (Newborn Infants)) OR (Newborn)) OR (Infant))) AND ((lung ultrasound score) OR (LUS))) AND (("Respiration, Artificial"[Mesh]) OR ((((Artificial Respiration) OR (Mechanical Ventilations)) OR (Mechanical Ventilation)) OR (Respirations, Artificial))). To expand the search scope, we also utilized the “related articles” function in Pubmed and searched the references of identified articles simultaneously. The included studies comprised both prospective and retrospective designs. Supplementary Table 1 provides a detailed description of the literature search strategy.

### 2.2 Inclusion and exclusion criteria

In line with the PRISMA 2000 framework, strict inclusion and exclusion criteria were applied in this analysis. Eligibility criteria for studies included the following conditions: (1) Studies focusing on patients in the newborn and early infancy stages; (2) Patients requiring mechanical ventilation; (3) Pulmonary ultrasound score used as an evaluation index of mechanical ventilation; (4) Availability of extractable multivariate logistic regression odds ratios (ORs) or standardized mean differences (SMD) and corresponding 95% confidence intervals (95% CI), or availability of sufficient data to calculate true positives, false positives, true negatives, and false negatives.

The following criteria led to study exclusion: (1) Studies based on animal models, systematic reviews, letters, or case reports; (2) Studies with non-extractable data.

### 2.3 Data extraction and quality assessment

Extraction of information from each eligible study included collecting the following details: the name of the first author, publication year, region of the population, study design, total number of patients, number of mechanical and non-mechanical ventilation patients, cut-off LUS value, and number of true positives, false positives, true negatives, and false negatives patients. Data extraction was carried out independently by two reviewers, with discrepancies addressed through discussion and agreement; unresolved conflicts were referred to senior authors for a final decision. The Newcastle-Ottawa Scale (NOS) (17) served as the tool for assessing the quality of the selected studies, focusing on domains such as the exposed cohort, comparability, outcome measurement, outcome assessment, and cohort follow-up. The scale ranges up to 9 points, with studies scoring 6 or higher regarded as high quality.

### 2.4 Statistical method

To conduct the preliminary synthesis of ORs or SMD and their corresponding 95% CIs, we employed the “metagen” function available in the R “meta” package. Assessment of heterogeneity was conducted through the *I*^2^ and Cochrane's *Q* test, where *I*^2^ < 50% or *p* > 0.05 was interpreted as nonsignificant heterogeneity. A random-effects model was applied for all data analysis. The influence of each individual study was evaluated through sensitivity analysis using the “metainf” function, followed by data re-analysis after eliminating highly sensitive studies. Publication bias was evaluated through Egger's test and visual inspection of the funnel plot.

In addition, following the application of the random-effects model, a bivariate mixed-effects regression approach was employed to estimate pooled sensitivity (Se), specificity (Sp), positive and negative likelihood ratios (pLR and nLR), as well as the diagnostic odds ratio (DOR), each with corresponding 95% confidence intervals. A summary receiver operating characteristic (SROC) curve was generated, and the area under the curve (AUC) was determined. Heterogeneity due to non-threshold effects was evaluated using the *I*^2^ statistic, while Deeks' funnel plot was utilized to detect publication bias and potential small-study effects. All statistical analyses were primarily performed using Stata 15.0 and RevMan 5.4.1.

## 3 Results

### 3.1 Identification of relevant studies

**Figure 1:**
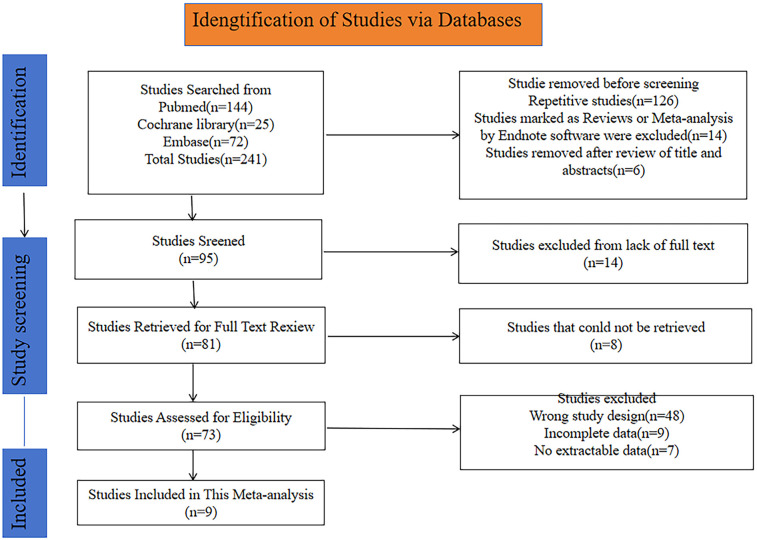
Flowchart for selection of studies included in this meta-analysis based on PRISMA guidelines.

A total of 241 potentially relevant articles were initially retrieved from the PubMed, Cochrane Library and Embase databases. After excluding duplicates and ineligible studies, 95 studies remained. Further screening resulted in the exclusion of 15 studies due to the unavailability of the full text. Additionally, 8 studies were removed as they could not be retrieved, leaving 73 studies for eligibility assessment. The following reasons led to the exclusion of certain studies: wrong study design (*n* = 48), incomplete data (*n* = 9), and no extractable data (*n* = 6). Ultimately, 9 studies with a total of 1,746 patients were incorporated in this meta-analysis. The flowchart of study selection and screening is depicted in Figure 1.

### 3.2 Study characteristics and quality assessment

Among the nine eligible studies, six had a total score ranging from 0 to 18, while the remaining three scored between 0 and 36. Regarding study design, seven were conducted prospectively, and two followed a retrospective approach. Among the included papers, two were from China, two from Italy, two from Spain, two from India, and one from Turkey. Six of the studies focused on newborns, and two on infants. The maximum age was less than 6 months for newborns, while the minimum age was for preterm infants less than or equal to 34 weeks. A total of 1,746 participants were enrolled across the included studies, comprising 854 males and 892 females. Among the included studies, four proposed lung ultrasound (LUS) cut-off for mechanical ventilation. Zhang et al. reported that infants <32 + 0 weeks' gestation with LUS >8 and those 32 weeks to 36 weeks and 6 days with LUS >7 predicted mechanical ventilation (18). Martini et al. found that LUS ≥ 11 in infants ≤34 weeks' gestation predicted MV requirement (19). De Rose et al. Found LUS >13 as a significant predictor (20). Pang et al. set the LUS cut-off for mechanical ventilation at 25.5 (21).

Quality evaluation conducted using the Newcastle-Ottawa Scale (17) revealed that each study scored above 6 points, indicating a generally high methodological quality and a reduced likelihood of bias. Detailed characteristics and quality ratings of the studies are summarized in Tables 1, 2.

**Table 1:** Characteristics and extracted data from included studies.

Author	Year	Study period	Country	Study design	Population	LUS score	No. of patients	Gender		Mean/Median age	Gestational age	LUS cut-off	Quality score
								Male	Female				
Vc et al. (16)	2024	2021/7–2023/6	India	Prospective	Within the first 24 h of birth	A total score between 0 and 18	192	130	62	2,306 ± 621g	35 ± 2.7 weeks		8
Pathak et al. (22)	2024	2020/8–2021/9	India	Prospective	Neonates	A total score between 0 and 18	65	41	24	1,540 ± 659g	32.4 ± 3. 7 weeks		7
Aliyev et al. (23)	2023	2018/1–2023/1	turkey	Retrospective	Infants born before 32 weeks	A total score between 0 and 18	218	110	108				7
Zhang et al. (18)	2023	2019/7–2021/4	China	Prospective	Premature infants	A total score between 0 and 36	857	365	492			7 and 8	7
Martini et al. (19)	2023	2020/5–2021/1	Italy	Prospective	Gestational age (GA) ≤ 34 weeks	A total score between 0 and 18	64	29	35	1,304 ± 502	30.3 ± 2.7 weeks	11	8
De Rose et al. (20)	2023	2022/10–2023/3	Italy	Retrospective	Neonates and infants aged <3 months	A total score between 0 and 36	60	32	28	3,141 ± 505	38 ± 0.7 weeks	13	7
Bobillo-Perez et al. (24)	2021	2019/10–2020/2	Spain	Prospective	Infants less than 6 months of age	A total score between 0 and 18	80	37	43	4.9 ± 1.5kg	53 (29–115) day		7
Gregorio-Hernandez et al. (25)	2020	2018/2–2029/3	Spain	Prospective	Infants under the gestational age of 35 weeks	A total score between 0 and 18	64	34	30				8
Pang et al. (21)	2019	2017/1–2018/1	China	Prospective	Newborns	A total score between 0 and 36	146	76	70			25.5	8

**Table 2:** Quality evaluation of the eligible studies with Newcastle–Ottawa scale.

Study	Selection				Comparability		Outcome		
	Representative-ness	Selection of non-exposed	Ascertainment of exposure	Outcome not present at start	Comparability on most important factors	Comparability on other risk factors	Assessment of outcome	Long enough follow-up (median≥72 h)	Adequacy (completeness) of follow-up
Vc et al. (16)	*	*	*	*	*	–	*	*	*
Pathak et al. (22)	*	*	*	*	–	–	*	*	*
Aliyev et al. (23)	–	*	*	*	*	–	*	*	*
Zhang et al. (18)	*	*	*	*	–	–	*	*	*
Martini et al. (19)	–	*	*	*	*	–	*	*	*
De Rose et al. (20)	*	*	*	*	*	–	*	–	*
Bobillo-Perez et al. (24)	–	*	*	*	*	–	*	–	*
Gregorio-Hernandez et al. (25)	–	*	*	*	*	–	*	*	*
Pang et al. (21)	–	*	*	*	*	–	*	*	*

*, indicates criterion met; –indicates significant of criterion not met.

### 3.3 Preliminary studies synthesis

**Figure 2:**
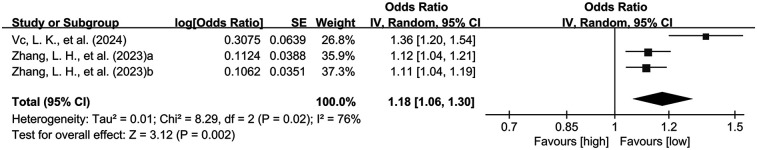
Forest plot of preliminary studies synthesis. OR, odds ratio; SE, standard error. Red squares represent the point estimates of the OR of each study, with 95% CI indicated by horizontal bars. Black diamond represent the summary estimate from the pooled studies with 95%CI.

The combined analysis of odds ratios (ORs) and their 95% confidence intervals (CIs) from the selected studies demonstrated that elevated LUS values increased the likelihood of requiring mechanical ventilation in neonates and infants (OR: 1.18, 95% CI: 1.06–1.30), with significant heterogeneity (*I*^2^ = 76%, *p* < 0.05), using a random-effects model (Figure 2).

**Figure 3:**
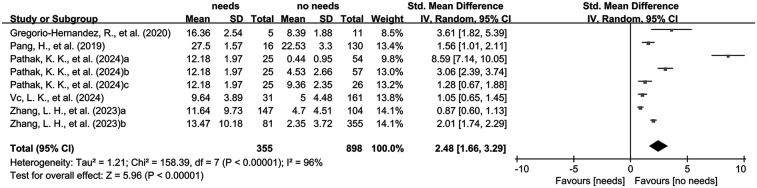
The pooled analysis of SMD and their corresponding 95% CIs extracted from the included studies.

The combined analysis of standardized mean differences (SMDs) and their 95% confidence intervals (CIs), derived from the included studies, revealed that the LUS of neonates and infants requiring mechanical ventilation was significantly higher than that of those without mechanical ventilation (SMD: 2.48, 95% CI: 1.66–3.29), with significant heterogeneity (*I*^2^ = 96%, *p* < 0.00001), using a random-effects model (Figure 3).

### 3.4 Sensitivity analysis

The sensitivity analysis aimed to evaluate the robustness of the results under hypothetical scenarios and to preliminarily explore potential sources of heterogeneity among the studies. A study was considered highly sensitive if its exclusion substantially reduced overall heterogeneity in the meta-analysis. No significant heterogeneity was observed in the initial synthesis, and no highly sensitive studies were identified.

### 3.5 Publication bias

**Figure 4:**
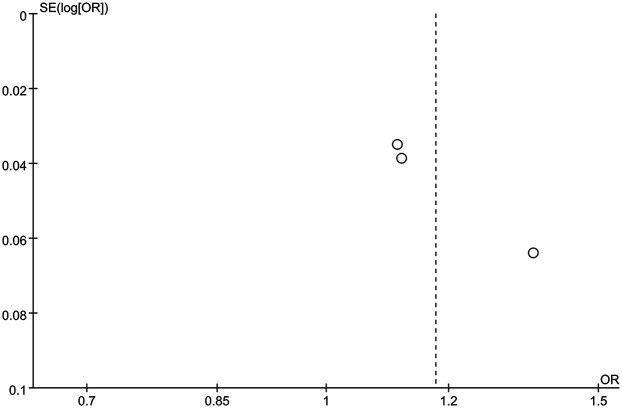
The funnel plot of categorical variables.

**Figure 5:**
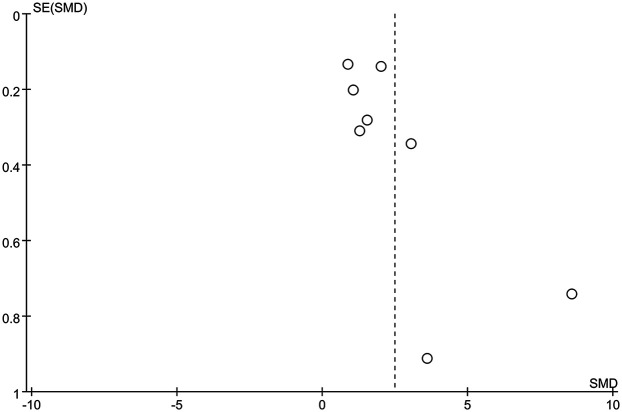
The funnel plot of continuous variables.

The funnel plot of categorical variables showed no publication bias (Figure 4). Egger's test results did not reveal any evidence of publication bias (*p* = 0.087). Similarly, the funnel plot of continuous variables showed no publication bias (Figure 5), and Egger's test confirmed this finding (*p* = 0.083).

### 3.6 Diagnostic data analysis results

**Figure 6:**
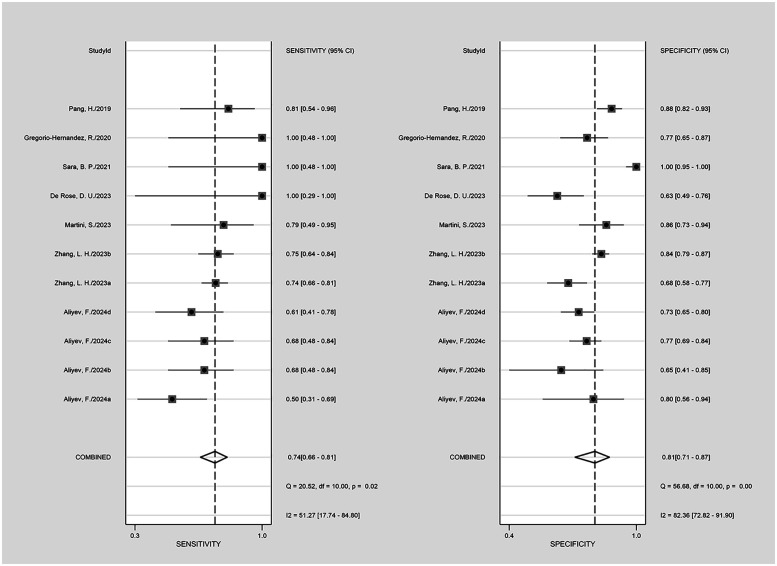
The forest map of the overall sensitivity, specificity, and CI confidence interval of lung ultrasound score for mechanical ventilation needs in the published research.

**Figure 7:**
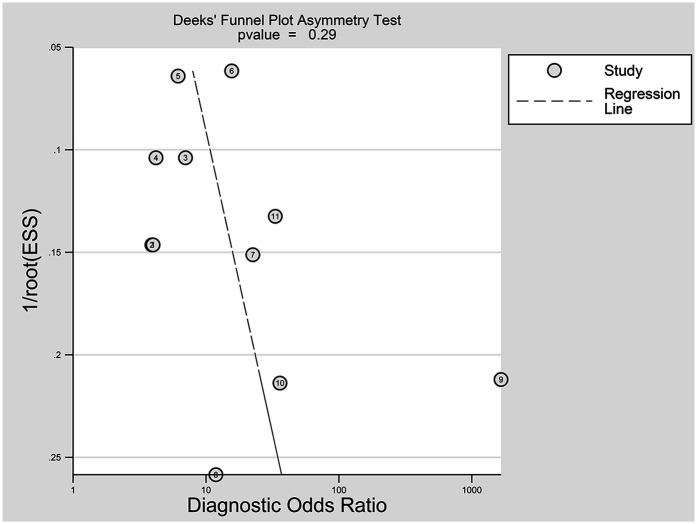
Egger test for publication bias. Egger test with a result of *p* = 0.29 illustrated that the publication bias of this meta-analysis was not obvious.

**Figure 8:**
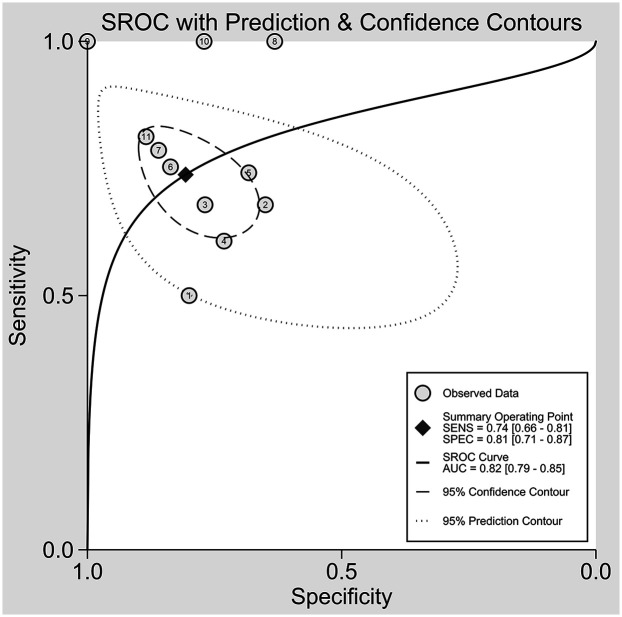
Funnel plot for sensitivity and specificity analysis. CI, confidence interval.

The nine included studies encompassed a total of 1,746 samples. LUS score predicted the need for mechanical ventilation, with overall sensitivity, specificity, positive likelihood ratio, negative likelihood ratio, and diagnostic odds ratio of 74% (95% CI: 66%–81%), 81% (95% CI: 71%–87%), 3.8 (2.4–6.1), 0.32 (0.23–0.46) and 12 (5–6), respectively. The overall forest plot showed significant heterogeneity in both sensitivity and specificity, with *p* = 0.81 and *I*^2^ = 82.36% (95% CI: 72.82%–91.90%) for sensitivity and *p* = 0.74 and *I*^2^ = 51.27% (95% CI: 17.74%–84.80%) for specificity (Figure 6). To evaluate publication bias, Deeks' funnel plot was utilized. The studies included in the analysis were symmetrically distributed around the regression line, and the *p*-value of 0.29 suggested the absence of significant publication bias (Figure 7). The SROC for all datasets demonstrated an AUC value of 0.82 (95% CI: 0.79%–0.85%) (Figure 8).

## 4 Discussion

As a non-invasive imaging evaluation tool, LUS can quantitatively grade the degree of lung lesions by systematically analyzing the characteristic ultrasound signs of the lungs (such as coalescent B lines, lung consolidation and pleural abnormalities) (26). The scoring system divides the bilateral chest into 12 standard areas and assigns a score ranging from 0 to 3 points according to the severity of abnormal signs in each area. The cumulative total score is positively correlated with the severity of lung lesions. Studies have shown that when the total LUS score exceeds a critical value, it often indicates that patients need mechanical ventilation support (27). In the field of critical care medicine, LUS has become an important auxiliary too for evaluating and managing patients requiring mechanical ventilation. The study by Pathak, et al. showed that LUS score correlates with different types of respiratory support and increases with the level of support (22). LUS scores have also been associated with clinical outcomes such as death, extubation failure, and recovery (28). In addition, this scoring system has important predictive value for clinical prognosis: a high LUS score is not only significantly correlated with mortality and the risk ofextubation failure in mechanically ventilated patients, but can also serve as an objective indicator to evaluate pulmonary inflammation resolution and to guide timing of weaning (29). These findings highlight the guiding role of LUS in individualized treatment decisions for critically ill patients.

In this meta-analysis, we found that higher LUS scores were correlated with an increased risk of mechanical ventilation in neonates and infants (OR: 1.18, 95% CI: 1.06–1.30). Neonates and infants requiring mechanical ventilation had significantly higher LUS scores than those who did not (SMD: 2.48, 95% CI: 1.66–3.29). Additionally, sensitivity analysis and Egger's test revealed no significant instability or publication bias among the included studies, further supporting the reliability of LUS in predicting the need for mechanical ventilation. The combined AUC value of LUS in predicting mechanical ventilation was 0.82 (95%CI: 0.79%–0.85%), indicating good diagnostic accuracy. Among the included studies, six used the 0–18 scoring system for pulmonary ultrasound, three used the 0–36 scale. Only four studies in the original literature reported LUS thresholds for mechanical ventilation with heterogeneous scoring methods, precluding determination of an optimal LUS cut-off for predicting mechanical ventilation. Current consensus recommends two LUS protocols for neonates and infants <1 year: (A) simplified (three regions per hemithorax-two anterior and one lateral-during the first 24–48 h) and (B) extended (five regions per hemithorax-similar to adults but with one lateral region-after 24–48 h) (30). Three included studies employed bilateral partitioning, a technique that complicates clinical application.

The previous meta-analysis by Razak et al. (31) included studies published prior to October 2018. It assessed the accuracy of LUS in predicting the need for surfactant therapy and mechanical ventilation in neonates with respiratory diseases receiving NCPAP support. Six studies were included: three used LUS scores, two utilized type 1 lung profiles, and one applied high-risk LUS assessment criteria. Their findings suggested that LUS could accurately determine whether neonates with respiratory distress on NCPAP required surfactant therapy or mechanical ventilation. In contrast, the present meta-analysis incorporates nine recent high-quality clinical studies, all of which used LUS scores. Through a combined analysis of multivariate categorical variables and diagnostic data, we found that a higher LUS score was positively associated with the need for mechanical ventilation, regardless of gestational age, including in young infants. Funnel plots and Deeks’ test indicated no publication bias among the included studies, further supporting the predictive value of LUS for mechanical ventilation in neonatal and infant populations.

The LUS score, derived from lung ultrasound, evaluates pulmonary lesions by quantifying several characteristic signs. As such, the LUS score can effectively predict the need for mechanical ventilation by reflecting the severity of pulmonary lesions and overall respiratory status and prognosis in children (32, 33). This study further confirmed the predictive value of high LUS scores for mechanical ventilation and suggests several underlying pathophysiological mechanisms: First, the vicious cycle of oxygenation dysfunction. A high LUS score directly reflects the extent of alveolar-interstitial edema and lung tissue consolidation by quantifying B-line density and the size of consolidated areas (26). Consolidated regions lose their capacity for gas exchange due to alveolar collapse and exudative filling. Coalescent B lines indicate interstitial edema, which thickens the alveolar-capillary barrier, exacerbating V/Q mismatch and leading to refractory hypoxemia (34). In such cases, it is difficult to correct hypoxia with conventional oxygen therapy, and positive end-expiratory pressure (PEEP) should be implemented through mechanical ventilation to reinflate and collapse alveolus, reduce intra-pulmonary shunt, and improve oxygenation efficiency through precise regulation of FiO₂ (35). Second, deterioration in respiratory mechanics and increased energy expenditure. Lung consolidation, resembling liver tissue (“hepatization”) due to organizing inflammatory exudate, markedly reduces lung compliance (36). In order to maintain the same tidal volume, the respiratory muscle of the child needs to overcome higher elastic resistance to do work, and the energy consumption increases. The high proportion of fatigue-prone type II fibers in children's diaphragm fibers makes them more prone to respiratory muscle fatigue (37). When the shallow respiratory index (RSBI) is >105 times/min/L or the maximum inspiratory pressure (MIP) is <30 cmH₂O, it indicates that the respiratory pump function is about to be decompensated, and mechanical ventilation can reduce oxygen consumption by partially or completely replacing spontaneous respiration, thereby preventing multi-organ failure (38). In addition, pulmonary ultrasound offers time-series early warning capability through dynamic monitoring, and LUS provides greater bedside accessibility and superior sensitivity for detecting parenchymal changes compared to chest x-ray (15). By establishing a LUS score trend model, it was found that an increase of ≥3 points within 24 h was associated with a 4.2-fold increase in the risk of intubation within 48 h (39). Typical warning signs include: progression from focal to coalescent on line B (40), dynamic air bronchogram within consolidation areas (indicating mechanization of exudation), and increased pleural effusion volume with disappearance of pulmonary slip sign. This real-time visual monitoring allows clinicians to implement lung-protective ventilation strategies before deterioration in blood gas analysis occurs (41).

This study has several limitations that may impact the conclusions and suggest directions for future improvement: (1) Limited number of included studies and the risk of potential selection bias: insufficient sample size may lead to reduced statistical efficiency and affect the robustness of the results. (2) Limited geographical representation and external validity: Most of the included studies were conducted in Asia and Europe, which may introduce confounding factors such as genetic differences, environmental exposures, and variations in clinical practice. This regional concentration limits the generalizability of the results to other populations. (3) Indicator heterogeneity affecting evidence quality: Significant heterogeneity was observed in key metrics, primarily due to variations in measurement tools, inconsistent follow-up periods, and differing definitions of clinical interventions. (4) Limited original data and inaccessible patient-level records preclude calculation of a precise cut-off, standardized LUS protocols and larger prospective studies are required to validate these findings (30).

## 5 Conclusions

The meta-analysis of multivariate categorical variables indicated that higher LUS scores were associated with an increased risk of mechanical ventilation in children. The combined diagnostic meta-analysis further demonstrated that LUS scores have high accuracy in predicting the need for mechanical ventilation. However, given the substantial heterogeneity among individual indicators and the presence of regional selection bias, there is a clear need to standardize primary endpoint assessment tools. Future efforts should focus on establishing an international, multicenter research platform and conducting large-scale prospective studies that include diverse populations, especially from underrepresented regions.

## Data Availability

The original contributions presented in the study are included in the article/Supplementary Material, further inquiries can be directed to the corresponding author.

## References

[1] Bresesti I Lista G. Respiratory support of neonate affected by bronchiolitis in neonatal intensive care unit. Am J Perinatol. (2020) 37(S 02):S10–3. 10.1055/s-0040-1713604 32898876

[2] Parkash A Haider N Khoso ZA Shaikh AS. Frequency, causes and outcome of neonates with respiratory distress admitted to neonatal intensive care unit, national institute of child health, Karachi. J Pak Med Assoc. (2015) 65(7):771–5. 26160089

[3] Mirza H Varich L Sensakovic WF Guruvadoo K Royall I Britt C Tracheomegaly among extremely preterm infants on prolonged mechanical ventilation. J Pediatr. (2020) 218:231–3.e1. 10.1016/j.jpeds.2019.10.024 31711760

[4] Torres-Castro C Valle-Leal J Martínez-Limón AJ Lastra-Jiménez Z Delgado-Bojórquez LC. Complicaciones pulmonares asociadas a ventilación mecánica en el paciente neonatal [pulmonary complications associated with mechanical ventilation in neonates]. Bol Med Hosp Infant Mex. (2016) 73(5):318–24. Spanish. 10.1016/j.bmhimx.2016.08.001 29384124

[5] Sovtic A Minic P Vukcevic M Markovic-Sovtic G Rodic M Gajic M. Home mechanical ventilation in children is feasible in developing countries. Pediatr Int. (2012) 54(5):676–81. 10.1111/j.1442-200X.2012.03634.x 22462757

[6] Choi YB Lee J Park J Jun YH. Impact of prolonged mechanical ventilation in very low birth weight infants: results from a national cohort study. J Pediatr. (2018) 194:34–9.e3. 10.1016/j.jpeds.2017.10.042 29198532

[7] Jensen EA DeMauro SB Kornhauser M Aghai ZH Greenspan JS Dysart KC. Effects of multiple ventilation courses and duration of mechanical ventilation on respiratory outcomes in extremely low-birth-weight infants. JAMA Pediatr. (2015) 169(11):1011–7. 10.1001/jamapediatrics.2015.2401 26414549 PMC6445387

[8] Yue G Wang J Li H Li B Ju R. Risk factors of mechanical ventilation in premature infants during hospitalization. Ther Clin Risk Manag. (2021) 17:777–87. 10.2147/TCRM.S318272 34354359 PMC8331080

[9] Rodriguez-Fanjul J Jordan I Balaguer M Batista-Muñoz A Ramon M Bobillo-Perez S. Early surfactant replacement guided by lung ultrasound in preterm newborns with RDS: the ULTRASURF randomised controlled trial. Eur J Pediatr. (2020) 179(12):1913–20. 10.1007/s00431-020-03744-y 32710304 PMC7378405

[10] Wang J Weng L Xu J Du B. Blood gas analysis as a surrogate for microhemodynamic monitoring in sepsis. World J Emerg Med. (2023) 14(6):421–7. 10.5847/wjem.j.1920-8642.2023.093 37969221 PMC10632753

[11] Nekoui M Seyed Bolouri SE Forouzandeh A Dehghan M Zonoobi D Jaremko JL Enhancing lung ultrasound diagnostics: a clinical study on an artificial intelligence tool for the detection and quantification of A-lines and B-lines. Diagnostics (Basel). (2024) 14(22):2526. 10.3390/diagnostics14222526 39594192 PMC11593069

[12] Raimondi F Yousef N Migliaro F Capasso L De Luca D. Point-of-care lung ultrasound in neonatology: classification into descriptive and functional applications. Pediatr Res. (2021) 90(3):524–31. 10.1038/s41390-018-0114-9 30127522 PMC7094915

[13] Staub LJ Biscaro RRM Maurici R. Emergence of alveolar consolidations in serial lung ultrasound and diagnosis of ventilator-associated pneumonia. J Intensive Care Med. (2021) 36(3):304–12. 10.1177/0885066619894279 31818178

[14] Wang Y Zhang Y He Q Liao H Luo J. Quantitative analysis of pleural line and B-lines in lung ultrasound images for severity assessment of COVID-19 pneumonia. IEEE Trans Ultrason Ferroelectr Freq Control. (2022) 69(1):73–83. 10.1109/TUFFC.2021.3107598 34428140 PMC8905613

[15] Wang G Ji X Xu Y Xiang X. Lung ultrasound: a promising tool to monitor ventilator-associated pneumonia in critically ill patients. Crit Care. (2016) 20(1):320. 10.1186/s13054-016-1487-y 27784331 PMC5081926

[16] Vc LK Patla VKR Vadije PR Murki S Subramanian S Injeti G Assessing the diagnostic accuracy of lung ultrasound in determining invasive ventilation needs in neonates on non-invasive ventilation: an observational study from a tertiary NICU in India. Eur J Pediatr. (2024) 183(2):939–46. 10.1007/s00431-023-05356-8 38052734

[17] Stang A. Critical evaluation of the Newcastle-Ottawa scale for the assessment of the quality of nonrandomized studies in meta-analyses. Eur J Epidemiol. (2010) 25(9):603–5. 10.1007/s10654-010-9491-z 20652370

[18] Zhang L Feng J Jin D Yu Z Qu Y Zheng M Lung ultrasound score as a predictor of ventilator use in preterm infants with dyspnea within 24 h after dhospitalization. Pediatr Neonatol. (2023) 64(4):420–7. 10.1016/j.pedneo.2022.09.019 36732096

[19] Martini S Gatelli IF Vitelli O Galletti S Camela F De Rienzo F Prediction of respiratory distress severity and bronchopulmonary dysplasia by lung ultrasounds and transthoracic electrical bioimpedance. Eur J Pediatr. (2023) 182(3):1039–47. 10.1007/s00431-022-04764-6 36562832

[20] De Rose DU Maddaloni C Martini L Ronci S Pugnaloni F Marrocco G Are lung ultrasound features more severe in infants with bronchiolitis and coinfections? Front Pediatr. (2023) 11:1238522. 10.3389/fped.2023.1238522 38161431 PMC10757344

[21] Pang H Zhang B Shi J Zang J Qiu L. Diagnostic value of lung ultrasound in evaluating the severity of neonatal respiratory distress syndrome. Eur J Radiol. (2019) 116:186–91. 10.1016/j.ejrad.2019.05.004 31153563

[22] Pathak KK Maria A Guleria M Mall PK Sharma A. Association of lung ultrasound scores with different modes of respiratory support and clinical outcomes: an observational study in a tertiary care neonatal unit. Cureus. (2024) 16(8):e66199. 10.7759/cureus.66199 39233940 PMC11373734

[23] Aliyev F Kayki G Annakkaya Kocyigit T İyigun İ Yigit S. Lung ultrasound scores within the first 3 days of life to predict respiratory outcomes. Pediatr Pulmonol. (2024) 59(3):662–8. 10.1002/ppul.26804 38131470

[24] Bobillo-Perez S Sorribes C Gebellí P Lledó N Castilla M Ramon M Lung ultrasound to predict pediatric intensive care admission in infants with bronchiolitis (LUSBRO study). Eur J Pediatr. (2021) 180(7):2065–72. 10.1007/s00431-021-03978-4 33585977

[25] Gregorio-Hernández R Arriaga-Redondo M Pérez-Pérez A Ramos-Navarro C Sánchez-Luna M. Lung ultrasound in preterm infants with respiratory distress: experience in a neonatal intensive care unit. Eur J Pediatr. (2020) 179(1):81–9. 10.1007/s00431-019-03470-0 31655870

[26] Mongodi S Chiumello D Mojoli F. Lung ultrasound score for the assessment of lung aeration in ARDS patients: comparison of two approaches. Ultrasound Int Open. (2024) 10:a24218709. 10.1055/a-2421-8709 39444846 PMC11497101

[27] Torres-Vargas C Legorreta-Soberanis J Sánchez-Gervacio BM Fernández-López PA Flores-Moreno M Alvarado-Castro VM Utility of a pulmonary oedema score for predicting the need for mechanical ventilation in COVID-19 patients in a general hospital. Arch Med Res. (2022) 53(4):399–406. 10.1016/j.arcmed.2022.03.006 35370011 PMC8938260

[28] Liu Y Zhou Y Liu P Ying W Wu H Dong Z. Combined lung and diaphragm ultrasound predicts extubation outcomes in ARDS: a prospective study. Eur J Med Res. (2024) 29(1):510. 10.1186/s40001-024-02103-z 39438932 PMC11495000

[29] Fu B Zhang P Zhang J. Diagnosis and prognosis evaluation of severe pneumonia by lung ultrasound score combined with Serum inflammatory markers. Mediterr J Hematol Infect Dis. (2023) 15(1):e2023057. 10.4084/MJHID.2023.057 38028392 PMC10631708

[30] Mongodi S Cortegiani A Alonso-Ojembarrena A Biasucci DG Bos LDJ Bouhemad B ESICM-ESPNIC international expert consensus on quantitative lung ultrasound in intensive care. Intensive Care Med. (2025) 51(6):1022–49. 10.1007/s00134-025-07932-y 40353867

[31] Razak A Faden M. Neonatal lung ultrasonography to evaluate need for surfactant or mechanical ventilation: a systematic review and meta-analysis. Arch Dis Child Fetal Neonatal Ed. (2020) 105(2):164–71. 10.1136/archdischild-2019-316832 31248960

[32] Ingelse SA Pisani L Westdorp MHA Almakdase M Schultz MJ van Woensel JBM Lung ultrasound scoring in invasive mechanically ventilated children with severe bronchiolitis. Pediatr Pulmonol. (2020) 55(10):2799–805. 10.1002/ppul.24974 32696620

[33] Oulego-Erroz I Del Pilar De Castro-Vecino M González-Cortés R Alonso-Ojembarrena A Rodríguez-Nuñez A Palanca-Arias D Lung ultrasound score, severity of acute lung disease and prolonged mechanical ventilation in children. Am J Respir Crit Care Med. (2024). 10.1164/rccm.202404-0843OC 39417699

[34] Patrician A Pernett F Lodin-Sundström A Schagatay E. Association between arterial oxygen saturation and lung ultrasound B-lines after competitive deep breath-hold diving. Front Physiol. (2021) 12:711798. 10.3389/fphys.2021.711798 34421654 PMC8371971

[35] Garberi R Ripa C Carenini G Bastia L Giani M Foti G Personalized ventilation guided by electrical impedance tomography with increased PEEP improves ventilation-perfusion matching in asymmetrical airway closure and contralateral pulmonary embolism during veno-venous extracorporeal membrane oxygenation: a case report. Physiol Rep. (2025) 13(7):e70280. 10.14814/phy2.70280 40214276 PMC11987203

[36] Bates JHT Smith BJ. Ventilator-induced lung injury and lung mechanics. Ann Transl Med. (2018) 6(19):378. 10.21037/atm.2018.06.29 30460252 PMC6212358

[37] Mantilla CB Greising SM Zhan WZ Seven YB Sieck GC. Prolonged C2 spinal hemisection-induced inactivity reduces diaphragm muscle specific force with modest, selective atrophy of type IIx and/or IIb fibers. J Appl Physiol (1985). (2013) 114(3):380–6. 10.1152/japplphysiol.01122.2012 23195635 PMC3568873

[38] Basile MC Mauri T Spinelli E Dalla Corte F Montanari G Marongiu I Nasal high flow higher than 60 L/min in patients with acute hypoxemic respiratory failure: a physiological study. Crit Care. (2020) 24(1):654. 10.1186/s13054-020-03344-0 33225971 PMC7682052

[39] Lukannek C Shaefi S Platzbecker K Raub D Santer P Nabel S The development and validation of the score for the prediction of postoperative respiratory complications (SPORC-2) to predict the requirement for early postoperative tracheal re-intubation: a hospital registry study. Anaesthesia. (2019) 74(9):1165–74. 10.1111/anae.14742 31222727 PMC7424553

[40] Mongodi S Stella A Orlando A Mojoli F. B-lines visualization and lung aeration assessment: mind the ultrasound machine setting. Anesthesiology. (2019) 130(3):444. 10.1097/ALN.0000000000002522. Erratum in: *Anesthesiology*. (2020) 132(6):1619. doi: 10.1097/ALN.0000000000003267 30664058

[41] Ramos Hernández C Núñez Delgado M Botana Rial M Mouronte Roibás C Leiro Fernández V Vilariño Pombo C Validity of lung ultrasound to rule out iatrogenic pneumothorax performed by pulmonologists without experience in this procedure. Rev Clin Esp. (2021) 221(5):258–63. English, Spanish. 10.1016/j.rce.2020.01.012 33998511

