# Supplementary material for: The predictive value of newborn and infant lung ultrasound score for mechanical ventilation needs: a systematic review and meta-analysis
Source: Front Pediatr. 2025 Oct 2;13:1642202. doi: 10.3389/fped.2025.1642202 (PMC12527875; doi:10.3389/fped.2025.1642202)
Supplement: Supplementary file 1 [file Table1.docx]

| Table S1 Detailed description of the literature search strategy | | |
| --- | --- | --- |
| **Databases** | **Literature search strategy** | Numberof literature |
| **Pubmed** | **((("Infant, Newborn"[Mesh]) OR ((((((((Infants, Newborn) OR (Newborn Infant)) OR (Newborn Infants)) OR (Neonate)) OR (Neonates)) OR (Newborns)) OR (Newborn)) OR (Infant))) AND ((lung ultrasound score) OR (LUS))) AND (("Respiration, Artificial"[Mesh]) OR (((((((Artificial Respiration) OR (Artificial Respirations)) OR (Respirations, Artificial)) OR (Ventilation, Mechanical)) OR (Mechanical Ventilations)) OR (Ventilations, Mechanical)) OR (Mechanical Ventilation)))** | 148 |
| **Embase** | 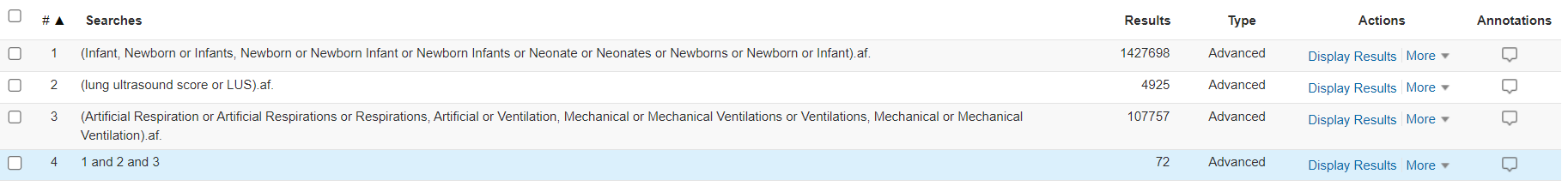 | 72 |
| Cochrane | 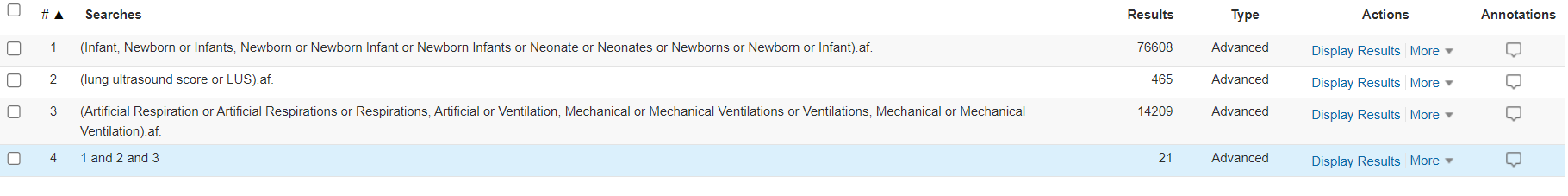 | 21 |
| web-of-science | **(Newborn Infants)) OR (Neonate)) OR (Neonates)) OR (Newborns)) OR (Newborn)) OR (Infant))) AND ((lung ultrasound score) OR (LUS))) AND ((Respiration, Artificial) OR (((((((Artificial Respiration) OR (Artificial Respirations)) OR (Respirations, Artificial)) OR (Ventilation, Mechanical)) OR (Mechanical Ventilations)) OR (Ventilations, Mechanical)) OR (Mechanical Ventilation)))** (Topic) | 72 |

Supplementary Material
